# Mantle hydration along outer-rise faults inferred from serpentinite permeability

**DOI:** 10.1038/s41598-017-14309-9

**Published:** 2017-10-24

**Authors:** Kohei Hatakeyama, Ikuo Katayama, Ken-ichi Hirauchi, Katsuyoshi Michibayashi

**Affiliations:** 1 Department of Earth and Planetary Systems Science, Hiroshima University, Higashi-Hiroshima, 739-8526 Japan; 2 Department of Geosciences, Faculty of Science, Shizuoka University, 836 Ohya, Suruga-ku, Shizuoka, 422-8529 Japan; 3 Institute of Geosciences, Shizuoka University, Shizuoka, 422-8529 Japan

## Abstract

Recent geophysical surveys indicate that hydration (serpentinization) of oceanic mantle is related to outer-rise faulting prior to subduction. The serpentinization of oceanic mantle influences the generation of intermediate-depth earthquakes and subduction water flux, thereby promoting arc volcanism. Since the chemical reactions that produce serpentinite are geologically rapid at low temperatures, the flux of water delivery to the reaction front appears to control the lateral extent of serpentinization. In this study, we measured the permeability of low-temperature serpentinites composed of lizardite and chrysotile, and calculated the lateral extent of serpentinization along an outer-rise fault based on Darcy’s law. The experimental results indicate that serpentinization extends to a region several hundred meters wide in the direction normal to the outer-rise fault in the uppermost oceanic mantle. We calculated the global water flux carried by serpentinized oceanic mantle ranging from 1.7 × 10^11^ to 2.4 × 10^12^ kg/year, which is comparable or even higher than the water flux of hydrated oceanic crust.

## Introduction

It has been observed that hydration of oceanic plate occurs in outer-rise regions through bending-related faulting prior to subduction. For example, seismic wide-angle refraction studies reveal that seismic velocities of crust and upper mantle are markedly reduced at outer-rise regions in the middle American Trench^1–3^, the Chile Trench^4,5^, the Kurile Trench^6^, the northern Japan Trench^7^, the Tonga‒Kermadec Trench^8^ and the Alaska Peninsula^9^. Serpentinite that forms by the hydration of oceanic mantle carries 13 wt% water to the deep mantle, and can remain stable down to 200 km depth in cold subduction zones^10^. Then, water is released into overlying mantle wedge by a dehydration reaction and the liberated water can induce partial melting and the formation of a magmatic arc^11^. In addition, intermediate-depth earthquakes occur in the lower plane of the double seismic zones, located within the subducting oceanic mantle, and these events might be triggered by serpentine dehydration reactions through dehydration embrittlement^12^. Thus, slab mantle hydration (serpentinization) can have a significant influence on the flux of subducting water and the mechanisms of earthquakes.

Yet there is still no consensus regarding the hydration processes that affect oceanic mantle, although water could penetrate along outer-rise faults that cut through the oceanic crust, thereby reaching the oceanic mantle and promoting serpentinization^13,14^. Numerical experiments have shown that stress changes induced by the bending oceanic plate produce sub-hydrostatic or even negative pressure gradients along normal faults, favoring downward pumping of fluids^15^. At the peridotite-water reaction front, serpentinization could be controlled by slower processes either reaction kinetics or water access to the reaction front^16^. Because chemical reactions are geologically rapid at temperatures above 100 °C^17^, the fluid transport rate through existing serpentinite into reaction front seems to be a primary factor controlling serpentinization. Consequently, the permeability of serpentinite seems to play an important role in controlling the extent of hydration of the oceanic mantle along outer-rise faults. In this study, we measured the permeability of low-temperature serpentinites under confining pressure. The serpentinites were sampled at an accretionary prism (Mineoka Belt, Japan) and dredged from the deep seafloor (Parece Vela Basin, South Mariana Trench and Tonga Trench). We discuss the lateral extent of serpentinization and the subduction water flux transported into the Earth’s interior.

## Results

**Figure 1:**
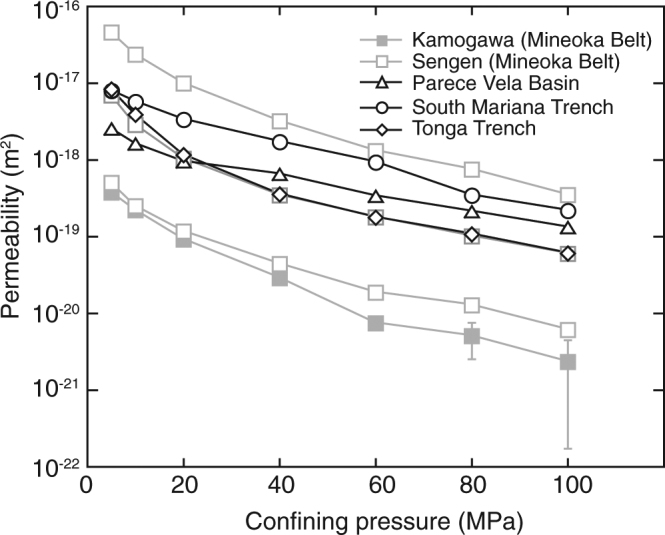
Experimental results showing the permeability of low-temperature serpentinites from the accretionary prism (Mineoka Belt) and deep seafloor (Parece Vela Basin, South Mariana Trench and Tonga Trench). Permeability varies between samples, but all samples show a similar pressure sensitivity with increasing confining pressure. Although most of the permeability represents intrinsic permeability after the correction for the Klinkenberg effect, the sample from the Kamogawa area has a very low fluid flux at high pressures (80 and 100 MPa), and the data represent gas permeability measured at 2 MPa of pore pressure, which may be slightly higher than the intrinsic permeability. Error bars of permeability are determined from uncertainly in flow-meter measurements.

The permeability of low-temperature serpentinites composed of lizardite and chrysotile was measured at a range of confining pressure (5–100 MPa). In all experiments, the permeability decreased with increasing confining pressure (Fig. 1, Supplementary Table S1), suggesting that the fluid conduits, including micro-cracks within specimens, are reduced with increasing confining pressure. Although samples from the accretionary prism show variable permeability (10^−21^ to 10^−19^ m^2^), the dredged serpentinites from the oceanic seafloor possess similar permeability (~10^−20^ m^2^) at a confining pressure of 100 MPa (Fig. 1, Supplementary Table S1). The permeability of low-temperature type serpentinite has higher values than that of high-temperature type serpentine (i.e., antigorite)^18,19^. The variable permeability in the accretionary prism may reflect variable alteration and micro-cracking after emplacement. The permeability of the dredged serpentinites is similar to that of basaltic rocks dredged from the Juan de Fuca and Tonga-Kermadec regions^20^. Differences in the permeabilities of samples from the accretionary prism can be correlated with the porosity of individual specimens, where samples with high porosity tend to have relatively higher permeability (Table 1, Supplementary Tables S1, S2). However, although the porosity of the dredged serpentinites is highly variable, ranging 10.5 to 24.7%, laboratory measurements show a narrow range of permeability in the dredged samples, suggesting that most of the pore spaces do not contribute to fluid flow. In fact, transport porosity, which is directly related to permeability, is reported to be much lower than bulk porosity, depending on pore geometry and connectivity^21,22^.

**Table 1:** Summary of experimental results.

Sample	Locality	Density (g/cm^3^)	Degree of serpentinization (%)	Porosity (%)	Permeability (m^2^)	Permeability pressure sensitivity (MPa^−1^)
Kamogawa	Mineoka Belt	2.72	90	0.3	2.3 × 10^−21^	2.9 × 10^−2^
Sengen-01	Mineoka Belt	2.50	100	—	6.0 × 10^−20^	2.7 × 10^−2^
Sengen-02	Mineoka Belt	2.46	100	0.0	6.1 × 10^−21^	2.8 × 10^−2^
Sengen-03	Mineoka Belt	2.48	100	6.6	3.5 × 10^−19^	3.3 × 10^−2^
KR03-D06-201	Parece Vela Basin	2.18	100	18.6	1.3 × 10^−19^	2.4 × 10^−2^
6K1364-R06	South Mariana Trench	2.43	87	10.5	2.2 × 10^−19^	3.6 × 10^−2^
6K1371-R26	Tonga Trench	2.17	100	24.7	6.1 × 10^−20^	2.7 × 10^−2^

Porosity and permeability at 100 MPa of confining pressure.

**Figure 2:**
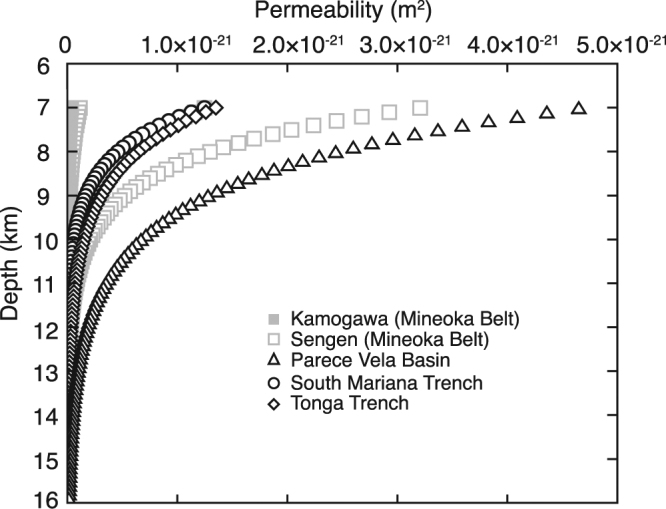
Depth dependent permeability of serpentinites based on the effect of pressure on permeability. Confining pressure is converted to depth using the average rock density of 2.8 g/cm^3^, and the depth is denoted below seafloor.

Although our experiments demonstrate permeability up to a confining pressure of 100 MPa, the pressure of the uppermost mantle exceeds 200 MPa. In applying the experimental results, we analyzed the relationship between permeability and pressure. The effect of pressure on permeability is determined using the following exponential functions^23^:

1 $$k={k}_{0}\exp [-\gamma (P-{P}_{0})]$$

where *k* is permeability, *P* is pressure, *γ* is pressure sensitivity, and subscript indicates the reference value. Most of the samples show a similar pressure dependence at relatively high confining pressures (>60 MPa); γ ranges from 2.4 × 10^−2^ to 3.6 × 10^−2^ MPa^−1^ (Table 1), which is comparable with other rock types^23^. Figure 2 and Table S3 shows the extrapolation of permeability with increasing depth that is converted by confining pressure, and the permeability of serpentinite under the conditions of the uppermost mantle located (7 km below the seafloor) is estimated in the order of 10^−23^ to 10^−21^ m^2^.

## Discussion

Although hydration processes in mantle rocks are complicated^24,25^, the flux of water delivery to the reaction front likely controls the extent of serpentinization because the chemical reactions that form serpentinite are geologically rapid at temperatures greater than 100 °C^16^. Fault damaged zones might act as a fluid pathways^26^, where water can infiltrate to great depths along the outer-rise faults. Given that the supplied water is immediately consumed by the formation of serpentinite, the water flux supplied to the reaction front likely controls the lateral extent of serpentinization. The water flux supplied from a fault to the reaction front (*q*_*fluid*_) can be expressed as follows, based on Darcy’s law:

2 $${q}_{fluid}=\frac{k}{\eta }\,\frac{dP}{L}$$

where *k* is permeability (m^2^), *dP* is pressure difference (Pa), *η* is fluid viscosity (Pa · s), and *L* is a distance from a fault to a reaction front (i.e., the lateral extent of serpentinization). The driving force for fluid flow is the pore fluid pressure difference (*dP*) between the hydrostatic pressure along a fault connected to the seafloor and equilibrium vapor pressure at the reaction front^16^. The time-integrated water flux (${Q}_{{fluid}}$) is then calculated as,

3 $${Q}_{fluid}=\int {q}_{fluid}dt$$

where *t* is the duration of water access. Given that the supplied water is consumed by the formation of serpentinite, the time-integrated water flux (*Q*_*fluid*_) is equivalent to the fluid mass of serpentinite (*Q*_*serp*_), which is expressed as follows:

4 $${Q}_{serp}=Lc\frac{{\rho }_{s}}{{\rho }_{w}}(1-\varphi )$$

where *L* is the lateral extent of serpentinization, *c* is the water content of serpentine, *ρ*_*w*_ and *ρ*_*s*_ are the densities of water and serpentine, respectively, and *ϕ* is the porosity. These relationships indicate that the lateral extent of serpentinization can be calculated from the duration of water access.

In these calculations, we used the temperature-dependent fluid viscosity, in which the thermal structure of the plate was computed from a one-dimensional heat conduction model^27^ with a plate age of 100 Myr (Supplementary Information). The water content of serpentine is 13 wt%, and the densities of water and serpentine are 1.00 g/cm^3^ and 2.55 g/cm^3^, respectively. The pore fluid pressure difference was estimated from the hydrostatic pressure and vapor pressure, and ranges from 117 to 166 MPa at depths of 7‒12 km.

**Figure 3:**
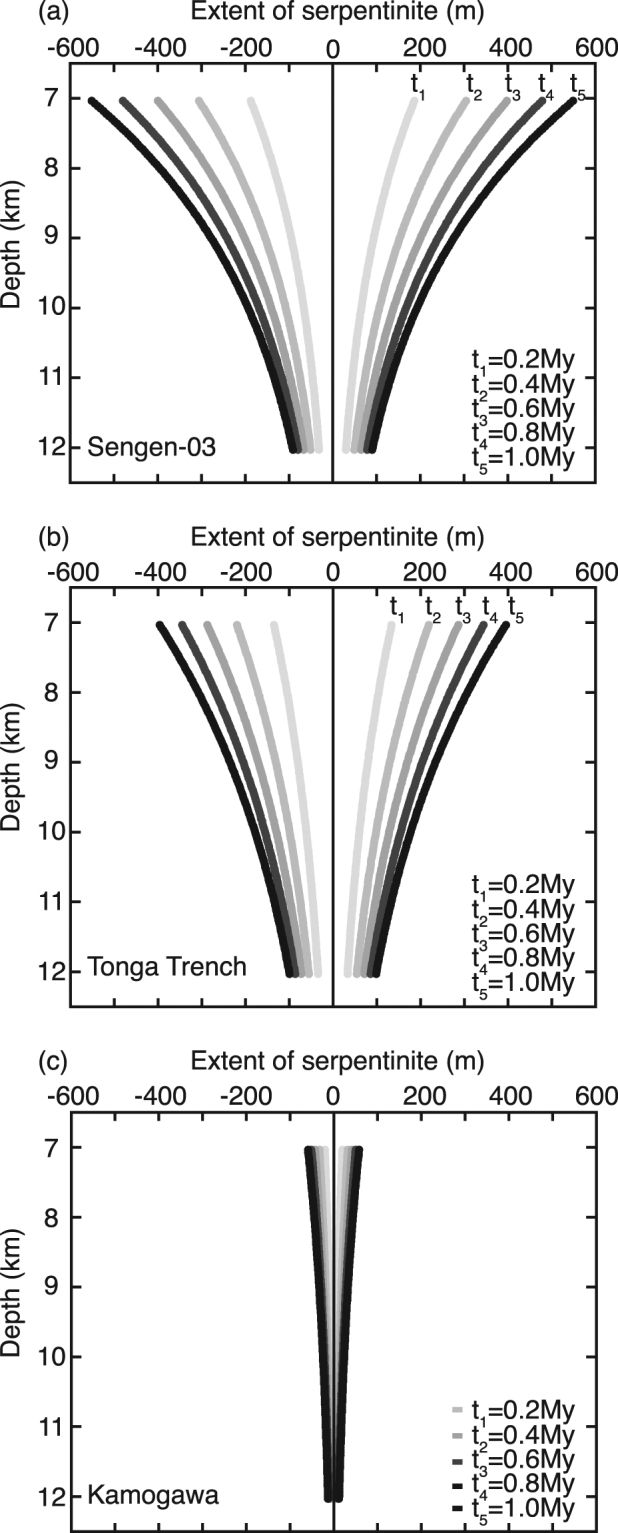
Extent of serpentinization along the outer-rise fault, showing the permeabilities of the Sengen-03 (**a**), the Tonga Trench (**b**), and the Kamogawa (**c**). The extent of serpentinization decreases with depth due to the effect of pressure on permeability. The calculation included the pressure-dependent permeability and porosity for Sengen-03 and Tonga Trench, whereas porosity is set to a constant value for Kamogawa because of the large uncertainty in the porosity measurements.

Figure 3 shows the calculated lateral extent of serpentinization based on the laboratory-determined permeability of three samples depending on the time scales of water availability. The highest permeability is from the Sengen area, the moderate permeability is from the Tonga Trench, and the lowest permeability is from the Kamogawa area. The lateral extent of serpentinization is relatively wider than below at shallow levels due to the higher permeability at lower pressures. Given that we assumed the water flux is the rate-limiting process, the lateral extent of serpentinization increases with time for water access through outer-rise faults. If the intermediate permeability is used for these calculations, and the time scale of water supply is assumed to be *t* = 1.0 Myr, serpentinization extends to a region 790 m wide in the direction normal to the outer-rise fault in the uppermost oceanic mantle (7 km depth below seafloor). The lateral extent tends to decrease with depth due to the effect of pressure on permeability, and it is limited to a width of 200 m at the tip of the fault zone (~12 km depth). Table 2 lists the extent of serpentinization calculated from the permeability of each sample. The permeability variations result in the wide range of lateral extents (120 to 1390 m), in the uppermost oceanic mantle at 7 km depth. The duration of water access depends on the convergence rates of the oceanic plate and the distance from the trench to the location of the outer-rise fault. If the duration of water access is between 0.2 and 1.0 Myr, the lateral extent of serpentinization for 1.0 Myr is about three times larger than that for 0.2 Myr (Table 2). We also calculated the lateral extent of serpentinization for a young and warm oceanic plate as old (20 Ma), which indicates that it experiences ~1.5 times the width of serpentinization of an old and cold plate owing the relatively low fluid viscosity of the old plate (Supplementary Information).

**Table 2:** Results of the numerical analysis of each sample.

Sample/Time scale	^1)^Extent of serpentinization (m)					^2)^Water content (wt%)	Subduction water flux (kg/year)
	1.0 Myr	0.8 Myr	0.6 Myr	0.4 Myr	0.2 Myr	1.0 Myr	1.0 Myr
^3)^Kamogawa	120	100	80	70	40	0.3	1.7 × 10^11^
^4)^Sengen-01	670	580	480	370	230	1.9	1.0 × 10^12^
^3)^Sengen-02	200	180	150	110	70	0.6	3.1 × 10^11^
Sengen-03	1100	960	800	610	370	2.8	1.5 × 10^12^
KR03-D06-201	1390	1210	1000	770	470	4.7	2.4 × 10^12^
6K1364-R06	700	610	510	390	240	1.6	8.5 × 10^11^
6K1371-R26	790	690	570	440	270	2.3	1.2 × 10^12^

^1)^The value is at 7 km depth.

^2)^Average water content in 5 km of uppermost mantle.

^3)^Calculated based on the constant porosity at 100 MPa.

^4)^Calculated based on the porosity of Sengen-02.

In the natural environments, the permeability of serpentinite can be modified with progressing the reaction of serpentinization, because serpentinization involves volume expansion^28^ that enhances fracturing in the rock specimen^16^. Although permeability at the serpentinized front is coupled to the chemical reaction, our experimental samples are nearly completed reaction (~100% serpentinization). However, even in the case of complete serpentinization, the samples show highly variable permeability, suggesting that the development of fractures plays an important role in fluid percolation and supply to the reaction front along the outer-rise faults.

**Figure 4:**
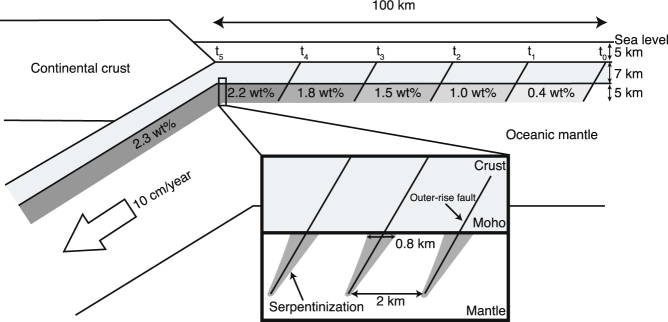
Schematic model of the hydration of oceanic lithosphere inferred from the permeability of the sample from the Tonga Trench. It is assumed that the thickness of the oceanic crust is 7 km, the depth of seawater is 5 km, the outer-rise faults occur at a distance of 100 km from the trench at 2 km intervals, and the fault depth is 12 km below the seafloor. The average water content increases towards the trench because the extent of serpentinization is a time-dependent process. The expanded inset shows the extent of serpentinization along an outer-rise fault, which is 0.8 km at 7 km depth.

Figure 4 shows a schematic hydration model of oceanic lithosphere at outer-rise region, illustrating serpentinization based on the intermediate permeability. Because a crack tends to be open in an extensional field related to plate bending, the water penetration rate within an outer-rise fault seems to be controlled by the permeability of fault damage zone, which may reach to 10^−14^ m^2^ ^26^, suggesting that pore pressure within faults zones is rapidly equilibrated to hydrostatic pressure. However, water infiltration along faults might not be effective in a compressional stress field, thereby raising the possibility that neutral-stress planes control a vertical water supply in the fault zones. In fact, the low-velocity anomalies at uppermost mantle in the middle American Trench are restrained to the depths of the normal-fault earthquake^29^.

Since outer-rise (extensional) earthquakes observed through earthquake focal mechanisms occur mainly at depths of <12 km and a distance of ca. 100 km from the trench^30^, water access along fault zones may be limited to the upper 5 km of the oceanic mantle, and the maximum time scale of water supply is ca. 1.0 Myr if the plate velocity is assumed to be 10 cm/year. Seismic reflection images reveal that the fault interval is observed at approximately 2 km^9,13^, and we used this interval in our calculations. Outer-rise earthquakes occasionally occur even at depths of >12 km. However, the transition in the stress field from extensional to compressional is located at 300–400 °C, which might limit the hydration of oceanic mantle^31^. The stress field also changes during a megathrust earthquake cycle^32^, meaning that mantle hydration may take place at greater depths after giant earthquakes, such as the 2011 Tohoku-oki earthquake. In this study, we assumed that water delivery controls the mantle hydration; however, hydration also affected by reaction kinetics, with the peak reaction temperature being in the range 270–300 °C^17^, suggesting that old oceanic lithosphere provides conditions that are favorable for serpentinization^33^.

Based on the distribution of serpentinite along an outer-rise fault calculated for the intermediate permeability, the subducting oceanic mantle contains 2.3 wt% water in the top 5 km of uppermost mantle. Table 2 lists the average water content for other samples, which range from 0.3 to 4.7 wt%. These estimates broadly agree with the water contents inferred from the perturbation of seismic velocity of the uppermost mantle beneath the Alaska Peninsula^9^ and beneath the Nicaragua region^4^. To estimate the global subduction water flux, we used the global subduction rate of oceanic crust (6.0 × 10^13^ kg/year)^34^. The results indicate the water flux carried by the serpentinized oceanic mantle range from 1.7 × 10^11^ to 2.4 × 10^12^ kg/year (Table 2). These ranges are comparable with the previous estimate of 7.9 × 10^11^ kg/year that assumes 2 wt% water is present in the top 5 km of the uppermost mantle^30^. We calculated the global subduction water flux assumed to hydrate the uppermost mantle to a depth of 15 km, and these results also are comparble to a the previous estimate^30^. Although our estimate has a large uncertainty, including the development of outer-rise faults and local variations in the oceanic lithosphere, the global fluid flux carried by the serpentinized oceanic mantle is comparable to that carried by the hydrated oceanic crust. For example, Bebout^34^ and Ito *et al*.^35^ calculated the water flux of the oceanic crust to range from 9.0 × 10^11^ to 1.8 × 10^12^ kg/year, assuming a water content of 1.5–3.0 wt% in the subducted oceanic crust; this range is comparable to the estimated water flux from hydrated oceanic mantle. Hacker^36^ proposed a water flux of the oceanic crust of 6.1 × 10^11^ kg/year, and calculated the subduction water flux of the oceanic mantle to be 5.7 × 10^11^ kg/year if the oceanic mantle is hydrated down to 4 km and contains 2 wt% water. Thus, the global water flux carried by serpentinized oceanic mantle is comparable to or higher than the water flux of hydrated oceanic crust.

Although geophysical observations such as seismic and magnetic surveys^37^ have identified mantle hydration along the outer-rise faults, these observations have a relatively low spatial resolution and cannot clearly determine the lateral extent of serpentinization along the fault plane. Our mantle hydration model based on serpentinite permeability proposes that highly localized serpentinization takes place along the fault planes, and this contributes to controlling the distribution of serpentinite along the outer-rise faults and estimating the subduction water budget.

## Method

### Samples

This study analyzed low-temperature serpentinites composed of lizardite and chrysotile, which are stable at the temperatures of uppermost oceanic mantle as low as 100 to 300 °C. The samples were collected from the accretionary prism at Kamogawa and Sengen located in the Mineoka Belt, Japan^38^, and from deep seafloor dredged from the Parece Vela Basin, South Mariana Trench and Tonga Trench^39,40^. Petrographic analyses are performed on thin sections, and degree of serpentinization is determined based on point counts of olivine, pyroxene and serpentine (Table 1). Samples from the Sengen, the Parece Vela Basin and the Tonga Trench are completely serpentinized, whereas the Kamogawa and the South Mariana Trench samples contain minor inherited olivine and pyroxene (degree of serpentinization, 87–90%). All samples consist mainly of lizardite, chrysotile, minor magnetite and calcite, and show mesh texture that is typical of low-temperature serpentinite (Supplementary Fig. S1). Cylindrical cored samples (20 mm in diameter) were prepared for measurements of permeability. To remove any absorbed water, the specimens were dried at 70 °C for several days prior to experiments. The density and degree of serpentinization of each specimen are listed in Table 1.

### Permeability and Porosity measurement

Intra-vessel deformation and fluid flow apparatus at Hiroshima University^41^ were used to measure the permeability of low-temperature serpentinites. Permeability was measured from the flow rate at a constant fluid pressure (P_P_ ≤ 2.0 MPa), where nitrogen gas was used as a pore fluid and the confining pressure was increased up from 5 to 100 MPa at room temperature. The steady-state fluid flux generated by the pore pressure gradient across the specimens was monitored using a digital flow meter, and gas permeability was calculated following Darcy’s law. The flow rate was determined using the equations of Kawano *et al*.^18^. Following this, the obtained gas permeability was converted to intrinsic permeability using the Klinkenberg effect^42^, which is not influenced by the type of pore fluid in the system. The experimental error on the permeability is mostly a function of the measurement accuracy of the fluid flux and the pore pressure dependence, which generally results in a <10% uncertainty in the estimate of intrinsic permeability, or a larger uncertainty in the case of extremely low fluid flux (i.e., permeability lower than 10^−20^ m^2^). We also measured permeability using water for some samples, and the results are approximately similar to those determined using the gas flow method. However, measurement of the steady-state fluid flow using water is technically difficult for the low-permeability samples and includes a large uncertainty in fluid flux due to the relatively high viscosity of water.

Porosity was also measured using the gas expansion method based on the isothermal (Boyle-Mariotte) gas equations, where the grain volume and pore volume of each specimen were measured using a gas porosimeter^41^.

### Data availability

All experimental data are included in this published article and its Supplementary Information files.

## Electronic supplementary material

Supplemental Information

## References

[1] Grevemeyer I, Ranero CR, Flueh ER, Kläschen D, Bialas J (2007). Passive and active seismological study of bending-related faulting and mantle serpentinization at the Middle America trench. Earth Planet. Sci. Lett..

[2] Ivandic M, Grevemeyer I, Bialas J, Petersen CJ (2010). Serpentinization in the trench-outer rise region offshore of Nicaragua: constraints from seismic refraction and wide-angle data. Geophys. J. Int..

[3] Van Avendonk HJA, Holbrook WS, Lizarralde D, Denyer P (2011). Structure and serpentinization of the subducting Cocos plate offshore Nicaragua and Costa Rica. Geochem. Geophys. Geosyst..

[4] Contreras-Reyes, E., Grevemeyer, I., Flueh, E. R. & Scherwath, M. Alteration of the subducting oceanic lithosphere at the southern central Chile trench-outer rise, *Geochemi. Geophys. Geosyst*. Q07003 (2007).

[5] Ranero CR, Sallares V (2004). Geophysical evidence for hydration of the crust and mantle of the Nazca plate during bending at the north Chile trench. Geology.

[6] Fujie G (2013). Systematic changes in the incoming plate structure at the Kuril trench. Geophys. Res. Lett..

[7] Fujie G, Kodaira S, Sato T, Takahashi T (2016). Along-trench variations in the seismic structure of the incoming Pacific plate at the outer rise of the northern Japan Trench. Geophys. Res. Lett..

[8] Contreras-Reyes, E. *et al*. Deep seismic structure of the Tonga subduction zone: Implications for mantle hydration, tectonic erosion, and arc magmatism, *J. Geophys. Res*. **116** (2011).

[9] Shillington DJ (2015). Link between plate fabric, hydration and subduction zone seismicity in Alaska. Nat. Geosci..

[10] Ulmer P, Trommsdorff V (1995). Serpentine stability to mantle depths and subduction‒related magmatism. Science.

[11] Kelley KA (2006). Mantle melting as a function of water content beneath back-arc basins. J. Geophys. Res..

[12] Peacock SM (2001). Are the lower planes of double seismic zones caused by serpentine dehydration in subducting oceanic mantle?. Geology.

[13] Ranero CR, Phipps Morgan J, McIntosh K, Reichert C (2003). Bending-related faulting and mantle serpentinization at the Middle America trench. Nature.

[14] Lefeldt M, Grevemeyer I, Goßler J, Bialas J (2009). Intraplate seismicity and related mantle hydration at the Nicaraguan trench outer rise. Geophys. J. Int..

[15] Faccenda M, Gerya VT, Burlini L (2009). Deep slab hydration induced by bending-related variations in tectonic pressure. Nat. Geosci..

[16] MacDonald AH, Fyfe WS (1985). Rate of serpentinization in seafloor environments. Tectonophysics.

[17] Martin B, Fyfe WS (1970). Some experimental and theoretical observations on the kinetics of hydration reactions with particular reference to serpentinization. Chem. Geol..

[18] Kawano S, Katayama I, Okazaki K (2011). Permeability anisotropy of serpentinite and fluid pathways in a subduction zone. Geology.

[19] Katayama I, Terada T, Okazaki K, Tanikawa W (2012). Episodic tremor and slow slip potentially linked to permeability contrasts at the Moho. Nat. Geosci..

[20] Christensen N, Ramananantoandro R (1988). Permeability of the oceanic crust based on experimental studies of basalt permeability at elevated pressures. Tectonophysics.

[21] Bernabé Y, Mok U, Evans B (2003). Permeability-porosity relationships in rocks subjected to various evolution processes. Pure Appl. Geophys..

[22] Yokoyama T, Takeuchi S (2009). Porosimetry of vesicular volcanic products by a water-expulsion method and the relationship of pore characteristics to permeability. J. Geophys. Res..

[23] David C, Wong TF, Zhu W, Zhang J (1994). Laboratory measurement of compaction-induced permeability change in porous rocks: Implications for the generation and maintenance of pressure excess in the crust. Pure Appl. Geophys..

[24] Iyer K, Austrheim H, Joun T, Jamtveit B (2008). Serpentinization of the oceanic lithosphere and some geochemical consequences: Constraints from the Leka Ophiolite Complex Norway. Chem. Geol..

[25] Okamoto A, Ogasawara Y, Ogawa Y, Tsuchiya N (2011). Progress of hydration reactions in olivine-H_2_O and orthopyroxenite-H_2_O systems at 250 °C and vapor-saturated pressure. Chem. Geol..

[26] Wibberley CAJ, Shimamoto T (2003). Internal structure and permeability of major strike-slip fault zones: the Median Tectonic Line in Mie Prefecture, Southwest Japan. J. Struct. Geol..

[27] Turcotte, D. L. & Schubert, G. *Geodynamics*, (ed. Turcotte, D. L. & Schubert, G.) 160‒229 (Cambridge Univ., 2002).

[28] O’Hanley DS (1992). Solution to the volume problem in serpentinization. Geology.

[29] Lefeldt M, Ranero CR, Grevemeyer I (2012). Seismic evidence of tectonic control on the depth of water influx into incoming oceanic plates at subduction trenches. Geochemi. Geophys. Geosyst..

[30] Emry EL, Wiens DA (2015). Incoming plate faulting in the Northern and Western Pacific and implications for subduction zone water budgets. Earth Planet. Sci. Lett..

[31] Contreras-Reyes E, Grevemeyer I, Fluech ER, Reichert C (2008). Upper lithospheric structure of the subduction zone offshore of southern Arauco peninsula, Chile, at ~38 °S. J. Geophys. Res..

[32] Obana K (2012). Normal-faulting earthquakes beneath the outer slope of the Japan Trench after the 2011 Tohoku earthquake: Implications for the stress regime in the incoming Pacific Plate. Geophys. Res. Lett..

[33] Iyer K, Rüpke LH, Morgan JP, Grevemeyer I (2012). Controls of faulting and reaction kinetics on serpentinization and double Benioff zones. Geochem. Geophys. Geosyst..

[34] Bebout, G. E. *Subduction Top to* Bottom (ed Bebout, G. E. *et al*.) 179‒193 (AGU, 1996).

[35] Ito E, Harris DM, Anderson AT (1983). Alteration of oceanic crust and geologic cycling of chlorine and water. Geochimi. Cosmochim. Acta.

[36] Hacker BR (2008). H_2_O subduction beyond arcs. Geochemi. Geophys. Geosyst..

[37] Naif S, Key K, Constable S, Evans RL (2015). Water-rich bending faults at the Middle America Trench. Geochem. Geophys. Geosyst..

[38] Ogawa Y, Takahashi A (2004). Seafloor spreading, obduction and triple junction tectonics of the Mineoka Ophiolite, Central Japan. Tectonophysics.

[39] Michibayashi K (2007). Variable microstructure of peridotite samples from the southern Mariana Trench: Evidence of a complex tectonic evolution. Tectonophysics.

[40] Michibayashia K, Harigane Y, Ohara Y, Muto J, Okamoto A (2014). Rheological properties of the detachment shear zone of an oceanic core complex inferred by plagioclase flow law: Godzilla Megamullion, Parece Vela back-arc basin, Philippine Sea. Earth Planet. Sci. Lett..

[41] Okazaki K, Noda H, Uehara S, Shimamoto T (2014). Permeability, porosity and pore geometry evolution during compaction of Neogene sedimentary rocks. J. Struct. Geol..

[42] Klinkenberg, L. J. The permeability of porous media to liquids and gases, *API Drill. Prod. Prac*. 200–213 (1941).

