# Supplementary material for: Mantle hydration along outer-rise faults inferred from serpentinite permeability
Source: Sci Rep. 2017 Oct 24;7:13870. doi: 10.1038/s41598-017-14309-9 (PMC5654952; doi:10.1038/s41598-017-14309-9)
Supplement: Supplementary file 1 — Supplemental Information [file 41598_2017_14309_MOESM1_ESM.pdf]

Supplementary Information

Mantle hydration along outer-rise faults inferred from serpentinite permeability

Kohei Hatakeyama[1]*, Ikuo Katayama[1], Ken-ichi Hirauchi[2] and Katsuyoshi Michibayashi[3]

[1]Department of Earth and Planetary Systems Science, Hiroshima University, Higashi-Hiroshima 739-8526, Japan

[2]Department of Geosciences, Faculty of Science, Shizuoka University, 836 Ohya, Suruga-ku, Shizuoka 422-8529, Japan

[3]Institute of Geosciences, Shizuoka University, Shizuoka 422-8529, Japan

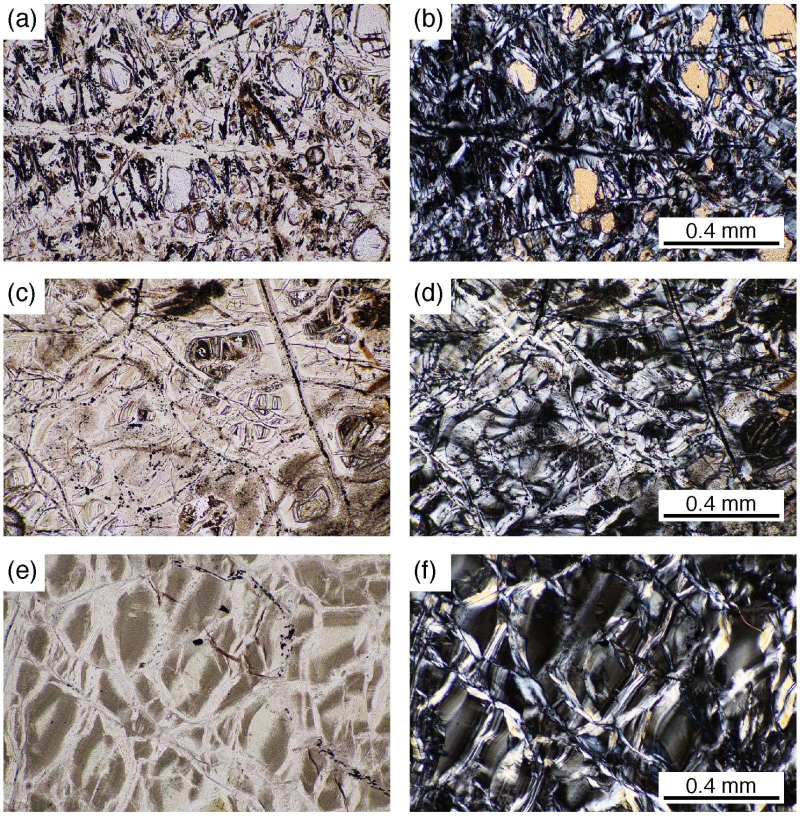

Figure S1. Microphotographs of mesh texture of low-temperature serpentinites from the South Mariana Trench (a,b), from the Tonga Trench (c,d) and from the accretionary prism in the Mineoka Belt (e,f). Left side images were taken under opened nicols (a,c,e) and right side images under crossed nicols (b,d,f). Serpentinites from the South Mariana Trench contain minor inherited olivine, and samples from the Tonga Trench and from the Mineoka Belt are completely serpentinized.

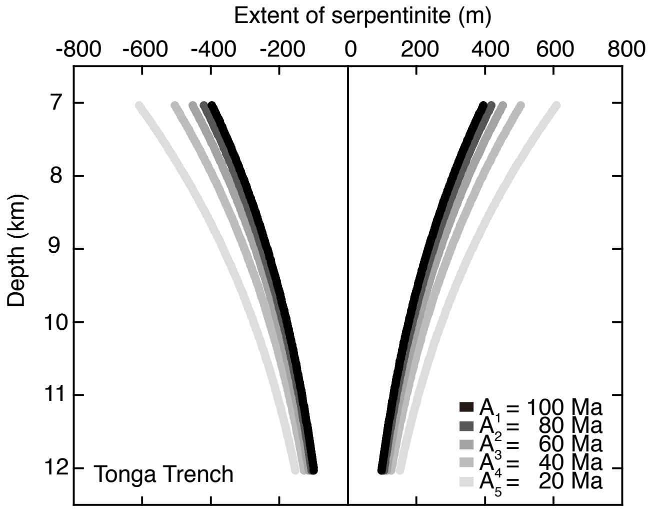

Figure S2. Effect of lithosphere age *A* on the lateral extent of serpentinization, showing the permeability of the Tonga Trench at a constant water supply duration of 1.0 Myr. The lateral extent of serpentinization involving young and warm lithosphere is wider than that of old and cold lithosphere owing to lower fluid viscosity.

Table S1. Results of permeability measurements

Sample / Confining pressure	5 MPa	10 MPa	20 MPa	40 MPa	60 MPa	80 MPa	100 MPa
				Intrinsic permeability (m^2)			
Kamogawa	3.8×10^{-19}	2.2×10^{-19}	9.2×10^{-20}	2.9×10^{-20}	7.4×10^{-21}	[1)] 5.6×10^{-21}	[1)] 2.3×10^{-21}
Sengen-01	6.9×10^{-18}	2.9×10^{-18}	1.0×10^{-18}	3.5×10^{-19}	1.8×10^{-19}	1.0×10^{-19}	6.0×10^{-20}
Sengen-02	5.0×10^{-19}	2.5×10^{-19}	1.2×10^{-19}	4.4×10^{-20}	1.9×10^{-20}	1.3×10^{-20}	6.1×10^{-21}
Sengen-03	4.6×10^{-17}	2.4×10^{-17}	9.9×10^{-18}	3.2×10^{-18}	1.3×10^{-18}	7.5×10^{-19}	3.5×10^{-19}
KR03-D06-201	2.5×10^{-18}	1.6×10^{-18}	9.4×10^{-19}	6.5×10^{-19}	3.4×10^{-19}	2.1×10^{-19}	1.3×10^{-19}
6K1364-R06	8.0×10^{-18}	5.8×10^{-18}	3.4×10^{-18}	1.7×10^{-18}	9.3×10^{-19}	3.5×10^{-19}	2.2×10^{-19}
6K1371-R26	8.3×10^{-18}	3.9×10^{-18}	1.2×10^{-18}	3.6×10^{-19}	1.8×10^{-19}	1.1×10^{-19}	6.1×10^{-20}

[1)] The data represent gas permeability measured at 2 MPa of pore pressure.

Table S2. Results of porosity measurements

Sample / Confining pressure	5 MPa	10 MPa	20 MPa	40 MPa	60 MPa	80 MPa	100 MPa
				Porosity (%)			
Kamogawa	0.6	0.5	0.5	0.4	0.3	0.3	0.3
Sengen-01	—	—	—	—	—	—	—
Sengen-02	1.3	1.2	0.9	0.6	0.3	0.1	0.0
Sengen-03	7.1	7.1	6.9	6.8	6.7	6.6	6.6
KR03-D06-201	21.4	21.2	20.9	20.3	19.8	19.4	18.6
6K1364-R06	11.3	11.2	11.1	10.9	10.8	10.7	10.5
6K1371-R26	26.2	25.9	25.8	25.4	25.2	25.0	24.7

Table S3. Results of extraplating permeability

Sample / Depth	7 km	8 km	9 km	10 km	11 km	12 km	13 km	14 km	15 km	16 km
					Extraplating peremability (m^2)					
Kamogawa	3.8×10^{-23}	3.7×10^{-23}	7.8×10^{-24}	3.5×10^{-24}	1.6×10^{-24}	7.2×10^{-25}	3.2×10^{-25}	1.5×10^{-25}	6.7×10^{-26}	3.0×10^{-26}
Sengen-01	1.2×10^{-21}	5.9×10^{-22}	2.8×10^{-22}	1.3×10^{-22}	6.3×10^{-23}	3.0×10^{-23}	1.4×10^{-23}	6.6×10^{-24}	3.1×10^{-24}	1.5×10^{-24}
Sengen-02	1.1×10^{-22}	5.4×10^{-23}	2.5×10^{-23}	1.3×10^{-23}	5.5×10^{-24}	2.5×10^{-24}	1.2×10^{-24}	5.5×10^{-25}	2.6×10^{-25}	1.2×10^{-25}
Sengen-03	3.2×10^{-21}	1.3×10^{-21}	5.3×10^{-22}	2.1×10^{-22}	8.6×10^{-23}	3.5×10^{-23}	1.4×10^{-23}	5.7×10^{-24}	2.3×10^{-24}	9.3×10^{-25}
KR03-D06-201	4.6×10^{-21}	2.4×10^{-21}	1.3×10^{-21}	6.7×10^{-22}	3.5×10^{-22}	1.8×10^{-22}	9.7×10^{-23}	5.1×10^{-23}	2.7×10^{-23}	1.4×10^{-23}
6K1364-R06	1.2×10^{-21}	4.6×10^{-22}	1.6×10^{-22}	6.3×10^{-23}	2.3×10^{-23}	8.5×10^{-24}	3.1×10^{-24}	1.2×10^{-24}	4.3×10^{-25}	1.6×10^{-25}
6K1371-R26	1.4×10^{-21}	6.5×10^{-22}	3.1×10^{-22}	1.5×10^{-22}	7.2×10^{-23}	3.4×10^{-23}	1.6×10^{-23}	7.9×10^{-24}	3.8×10^{-24}	1.8×10^{-24}

Relationship between age of the oceanic plate and lateral extent of serpentinization

Our mantle hydration model employs the temperature-dependent fluid viscosity, in which the temperature of the lithosphere T is computed from a half-space cooling model as follows:

$$T = T_1 - T_0 \left[erf\left(\frac{|y|}{2\sqrt{KA}}\right)\right] + T_0$$

where $T_1 - T_0 = 1300$ °C, y is the depth (m), K is the thermal diffusivity of the rock (10^{-6} m^2/s), T_0 is the surface temperature (0 °C), and A is the age of the lithosphere (s). The fluid viscosity η is dependent on temperature as follows:

$$\eta = \frac{0.1}{2.1482\left(T - 8.435 + \sqrt{8078.4 + (T - 8.435)^2}\right) - 120}$$

where T is temperature (°C). Thus, the lateral extent of serpentinization depends on the age of the lithosphere, with younger plates having a greater extent than that of older plates (Fig. S2). For example, if the age of the oceanic plate is 20 Ma, then the fluid viscosity at mantle depths is ca. 57 % lower than that for a plate with an age of 100 Ma and the lateral extent of serpentinization for a water supply duration of 1 Myr is greater by ca. 50 %.
